# Evaluation of utilisation and consequences of CRP point-of-care-testing in primary care practices: qualitative interviews with GPs from Germany

**DOI:** 10.3399/BJGPO.2024.0076

**Published:** 2025-03-12

**Authors:** Paul Jung, Jutta Bleidorn, Susanne Doepfmer, Christoph Heintze, Markus Krause, Lisa Kuempel, Doreen Kuschick, Lena-Sophie Lehmann, Liliana Rost, Kahina J Toutaoui, Florian Wolf

**Affiliations:** 1 Institute of General Practice and Family Medicine, Jena University Hospital, Friedrich Schiller University, Jena, Germany; 2 Institute of General Practice, Charité – Universitätsmedizin Berlin, Corporate Member of Freie Universität Berlin and Humboldt-Universität zu Berlin, Berlin, Germany

**Keywords:** C-reactive protein, point-of-care testing, primary healthcare, general practitioners

## Abstract

**Background:**

The use and advantages of point-of-care tests (POCTs) for C-reactive protein (CRP) in general practice, especially for upper respiratory tract infections (uRTIs), have been studied extensively. However, there is limited knowledge about test indications, prerequisites, and integration of these tests into everyday practice.

**Aim:**

To investigate the attitudes and experiences of GPs in Germany regarding the use of semi-quantitative C-reactive protein point-of-care tests (CRP-POCTs). The study places special emphasis on implementation in routine care, including testing procedures, feasibility, opportunities, and barriers for specific consultation scenarios, as well as test indications and their impact on GP–patient communication.

**Design & setting:**

Qualitative interview study with 10 GPs (May 2023–August 2023) in Germany.

**Method:**

Ten German GPs who participated in an observational study on CRP-POCT use in general practices were interviewed using semi-structured interviews. Audio-recordings were transcribed and content analysis was performed.

**Results:**

Interviewed GPs stated that CRP-POCTs offer several advantages for various treatment cases. The tests improve diagnostic confidence and certainty of GPs’ therapeutic decisions, and offer a broad spectrum of indications and application scenarios. Additionally, the tests have a positive impact on GP–patient communication, and their ease of use enables rapid implementation into existing workflows. On the other hand, CRP-POCTs increase the time required for test performance and patient consultation.

**Conclusion:**

Owing to the numerous benefits of semi-quantitative CRP-POCTs, interviewed GPs have a favourable attitude towards their regular integration into everyday practice. Implementation barriers include increased time and personnel expenses for testing and inadequate reimbursement by German statutory health insurance.

## How this fits in

Measurement of C-reactive protein (CRP) can give GPs a first impression of whether an infectious or inflammatory disease is present in the patient. Point-of-care tests (POCTs) can accelerate and support diagnostic decision making of GPs. Despite being widely used in many countries, C-reactive protein point-of-care tests (CRP-POCTs) are not commonly used in German general practices. This study presents qualitative data on indications, diagnostic goals, and clinical consequences of semi-quantitative CRP-POCTs in routine primary care. GPs find the use of CRP-POCTs easy to implement in daily routines and beneficial owing to positive effects on patient interaction.

## Introduction

Patient-near POCTs are particularly important in primary care. The results are available within a few minutes and the tests improve diagnostic as well as therapeutic decisions. Contrary to their diagnostic accuracy, the therapeutic consequences, feasibility, and successful integration into daily practice of POCTs are much less studied.^1–3^

POCTs measuring CRP can provide information on the extent of infectious diseases and can support the differentiation between bacterial and viral infections.^4^ Thus, CRP-POCTs support rational antibiotic prescribing in respiratory tract infections (RTIs) in primary care,^1,4–7^ and help to reduce antibiotic resistance.^8,9^

Previous publications have mainly focused on factors contributing to the use of CRP-POCTs in RTIs.^10–16^ There is limited evidence on the utilisation of CRP-POCTs by GPs for other conditions, such as gastrointestinal (GI) complaints or urinary tract infections (UTIs), and for monitoring therapy or disease progression. However, in outpatient emergency medical services, CRP-POCTS showed benefits for GI complaints and UTIs,^17^ but despite this finding only about 20% of GPs in Germany currently use CRP-POCTs,^18,19^ probably since reimbursement by statutory health insurance does not cover costs. However, the increasing workload of GPs and the need for adequate diagnostic tests in infectious diseases could be solved by an expansion of CRP-POCTs in the German primary care setting.

This study aimed to provide insights on general testing procedures, feasibility, and integration into practice workflows, test occasions, barriers and promoting factors for POCT use, and influence on GP–patient communication.

## Method

### Study design

This qualitative study was conducted as part of a prospective multicentre observational study with 1740 semi-quantitative CRP-POCTs performed in 49 German primary care practices, and was funded by the German Federal Ministry of Education and Research as part of the RESPoNsE practice-based research network.^20^ We conducted semi-structured interviews on CRP-POCT use with 10 GPs,^21,22^ who had participated in the observational study. The study followed the COnsolidated criteria for REporting Qualitative research (COREQ) (Supplementary Table 1).^23^

### Recruitment

Interview partners were recruited via personal, telephone, and email contacts. There were no other specific inclusion and exclusion criteria. Participants were provided with a financial compensation (EUR 100; approximately 85 GBP).

### Conducting the interviews

A guide for the semi-structured interviews and a questionnaire were developed by the researchers (Supplementary Boxes S1 and S2), considering domains and selected constructs of the updated Consolidated Framework for Implementation Research (CFIR)^24^ (Supplementary Table 2). Not all constructs of the different domains could be addressed owing to the extent of information. The proposed questions of the CFIR guide were not fully suitable for the objectives of our interviews. In addition, we had already gained insights from the data collection sheets (observational part of the study) and wanted to avoid redundancies in certain CFIR constructs. Yet, all five CFIR domains and several constructs were incorporated into the selection of our main categories. Two physicians piloted the interview guide. All interviews were conducted by the first author between May and August 2023, either face to face in practices or as online meetings.

### Data collection and processing

Audio-recordings were used for data collection. Field notes were taken immediately after each interview. No further information was obtained in the last three interviews (numbers 8 to 10), thus thematic saturation was assumed.^25^ Interviews were recorded, pseudonymised, and transcribed using F4X software. Transcripts were not returned for commenting or correction.

### Analysis

**Figure 1.**
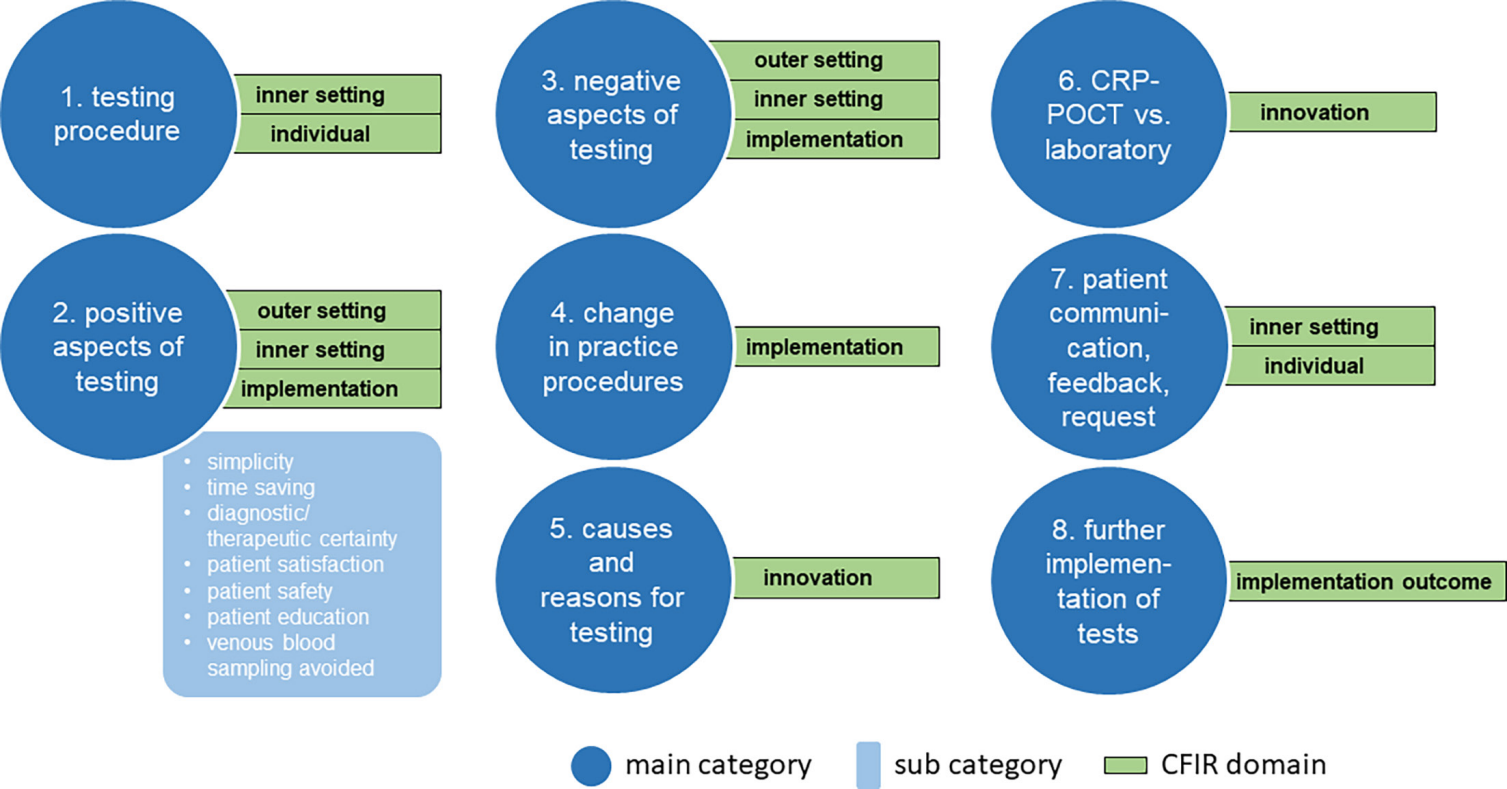
Illustration of the category system used. The eight main categories and exemplary subcategories for 'positive aspects of testing' in MAXQDA 2022 are shown. Matching Consolidated Framework for Implementation Research (CFIR) domains are shown in green. CRP-POCT = C-reactive protein point-of-care testing

Since the updated CFIR does not provide a template for evaluation with the software MAXQDA (version 2022),^26^ we performed a content analysis according to Kuckartz and Rädiker^27^ instead. Eight main categories were deductively derived from the interview guide, supplemented by inductively formed subcategories. The category system used is shown in Figure 1. Each text segment was assigned to a maximum of one subcategory per interview. For validity analysis, we calculated intra-coder and inter-coder reliability.^28^

## Results

Characteristics of the participants are shown in Table 1. Responders had between 2.5 years and 30 years of working experience as GPs, and worked in practices in Thuringia (*n* = 8) and Berlin (*n* = 2). The majority were female (*n* = 7). Interviews lasted between 12.47 minutes and 29.33 minutes. Seven GPs surveyed stated that they had not used CRP-POCTs within their practice before the observational study.

**Table 1.** Overview of the characteristics of the responders

Participant	Practice location according to BBSR^a^	Sex	Age, years	Work experience as GP, years	Location of the interview	Duration of the interview, min:sec
**P1**	Large city	Female	61	16	Practice	23:37
**P2**	Large city	Female	55	17	Practice	16:05
**P3**	Small town	Male	40	6	Practice	17:51
**P4**	Large city	Male	62	30	Online	29:33
**P5**	Medium-sized town	Female	37	2.5	Practice	18:45
**P6**	Medium-sized town	Female	52	20	Online	25:26
**P7**	Rural community	Male	37	3	Online	20:17
**P8**	Large city	Female	65	30	Practice	17:13
**P9**	Large city	Female	42	5	Online	15:37
**P10**	Large city	Female	55	13	Practice	12:47
		Female*: n* = 7 (70%) Male: *n* = 3 (30%)	Average: 50.6	Average: 14.25	Practice: *n* = 6 (60%) Online: *n* = 4 (40%)	Average:19:43

^a^Categories: rural community: <5 000 inhabitants; small town: 5 000–19 999 inhabitants; medium-sized town: 20 000–99 999 inhabitants; large city: ≥100 000 inhabitants. BBSR: Federal Institute for Research on Building, Urban Affairs and Spatial Development.

### Procedures for testing and modifying practice workflows

Conducted interviews showed that there were differences in CRP-POCT-use and procedures between practices. Both professions, GPs and medical assistants (MAs), decided, either individually or together, whether the test was carried out. Tests were never conducted solely by GPs, as MAs were always involved or carried out the tests independently. Turnaround time, including evaluation, ranged from 3–25 minutes.

Although almost all responders stated that the tests had '*not really brought any major changes in the practice workflows*‘ (P7), additional time was required whenever POCTs were performed:

*'Of course, I then had to see a patient a second or third time, which* [otherwise] *would not have been the case.* [...]*. The testing time prolonged the patient’s consultation*.' (P9)

Some responders also mentioned that planning for a follow-up appointment or avoiding it as a consequence of testing caused additional time expenditure. This contrasts with later mentions of time savings owing to accelerated test results and treatment decisions.

Most responders found it easy to integrate the tests into practice routines and workflows. The need for well-trained MAs and improvements in organisation, billing, and planning were also mentioned. One responder suggested that:

*'... you* [would] *have to organise it in such a way that right at the beginning of the consultation* [...] *if someone comes with an infectious disease* [...] *you already perform the test.‘* (P5)

### High costs and handling as negative aspects of tests

Initially, negative aspects as well as difficulties with testing, and barriers to regular use, were not explicitly addressed. When asked, the participants described the reimbursement as inadequate and some demanded simplified handling.

*'It was difficult to handle the pipette.‘* (P7)

In contrast to the rapid availability of test results, which was viewed positively, the additional staff and time required to perform the test in the practice were viewed negatively:

*'It is of course* [...] *an additional examination* [...]*. Practice time* [...] *is blocked and someone is busy.‘* (P1)

### Testing occasions and reasons

Patients' age and medical history were often considered when deciding to perform a CRP-POCT. Therefore, patients were categorised by the evaluators as high risk (aged >70 years, with multimorbidity and/or chronic illness), adult (aged >18 years and <70 years), and paediatric (aged ≤18 years). The primary purpose of CRP testing was to differentiate between bacterial and viral infections. Suspected areas of infection were mainly RTIs, occasionally UTIs. Symptoms that prompted testing included fever, unspecific complaints, poor general condition, high disease burden, or gastrointestinal complaints:

*'... infections, sometimes abdominal pain,* [...] *unclear symptoms, malaise* [...]*, poor general condition.* [We tested] *patients* [...] *with a previous illness, lung disease, COPD* [chronic obstructive pulmonary disease] *or asthma.‘* (P6)

Abnormal auscultation findings, cough, irritated mucosa, and swollen lymph nodes were mentioned at least once.

The tests were also conducted owing to organisational constraints such as limited laboratory availability on weekends:

*'Lab drivers come every day, but if* [...] *someone comes* [afterwards] *the result of a lab test would only be available days later.‘* (P4)

In these cases, diagnostic confidence was needed to clarify whether it was safe for the patients to delay further clinical intervention or if therapeutic measures should be initiated promptly.

### Benefits of tests in communication, diagnostic and therapeutic confidence, and patient safety and satisfaction

Tests were seen as useful for communicating with patients. For example, patients’ preferences for or against antibiotics could be discussed more specifically, and a (non-)prescription could be justified more easily.

*'For patients who* [...] *wanted an antibiotic it was good for communication. I could state: "We have no evidence of a bacterial infection* [...] *the antibiotic* [makes] *no sense.*"' (P2)

Based on better communication there were also reported increases in patient compliance. All of the interviewees stated at least once that testing had benefits on their diagnostic and therapeutic confidence. They used it *‘sometimes simply to confirm the first impression. So the test is not only organisationally preferable, but also when I say I'm not sure'* (P9).

Additionally, the tests offered satisfaction to the patients: *‘They found* [...] *it really great* [...] *that we could examine them and say this is bacterial, this is viral.’* (P5).

Responders also reported a direct impact on patient safety: ‘*There were two hospital admissions, which we, did more quickly than we might otherwise have done.'* (P10). The time-savings that resulted from the fast test results were also commented on by other responders:

*'The speed of the result, that’s fantastic.* […] *It was great having a result straight away and to be able to react immediately.‘* (P5)

### Patient feedback and clarification of tests

Before testing, GPs often explained the background, significance, and limitations of the rapid test to their patients. However, patients were rarely involved in the decision to undergo POC testing. The GP’s assessment and the expected benefits for subsequent diagnosis and treatment were primary considerations:

*'It’s difficult to leave freedom to decide. If I* [...] *come to the conclusion that I need the* [... test]*, then I'll do it.'* (P7)

After testing, however, there was '*always* [...] *positive feedback‘* (P2).

### Further implementation of the tests

All GPs interviewed stated that they could imagine using CRP-POCTs beyond the scope of the study:

*'I continue using them because they are simple, integration is practised, and they give quick information — you have a decision within a quarter of an hour.’* (P7)

Some responders mentioned using the tests for home visits and out-of-hours outpatient services. Two GPs already introduced quantitative automated CRP-POCTs in their practice because of the many advantages in general. However, semi-quantitative POCTs are seen as a useful alternative, for example, during home visits, as the trend of the value can often provide a basis for further assessment:

*'Organisationally, it is also quite exciting when someone just wants a follow-up check to see if the CRP is going down* [...]. *It would be good to have more precise numbers, quantitatively. But it was actually sufficient to have a rough direction.'* (P7)

## Discussion

### Summary

This qualitative interview study conducted with 10 German GPs from Thuringia (*n* = 8) and Berlin (*n* = 2) indicates that semi-quantitative CRP-POCTs can be easily integrated into daily practice, and add value to the diagnosis and management of patients with various types of infections. Utilisation of the test promotes patient communication and has a time-saving effect on the duration of treatment in practice. However, barriers of adoption include the ease of use of the CRP-POCT and inadequate reimbursement.

Overall, CRP-POCTs were perceived as beneficial owing to their feasibility and the rapid availability of results. Their use improved communication with patients regarding indication and performance, and allowed for information to be gained for further diagnosis and treatment, leading to enhanced treatment safety for GPs and patients. The testing procedure varied between practices in terms of personnel and time. However, testing was generally delegated as none of the GPs interviewed reported performing the tests in person.

### Strengths and limitations

The interviews were conducted shortly after the completion of the observational study, which helped to minimise recall bias and allowed interviewed GPs to report on actual experiences. Data analysis showed high levels of both, inter-coder (kappa according to Brennan and Prediger = 0.88) and intra-coder reliability (0.96), indicating valid interpretation. Other strengths are the direct mapping of physicians’ opinions regarding the utilisation of the tests, the identification of benefits and barriers, and the presentation of substantive, inductively collected ratings. Not all CFIR constructs could be integrated into our category system, but care was taken to ensure that all five CFIR domains were proportionally reflected in our main and subcategories (Figure 1).

There are few limitations to our study. Regarding the sampling, it is important to note that participants were pre-selected, which may have positively influenced the results. Although the interviewees were informed that their answers were pseudonymised, it is still possible that social desirability bias may have influenced their responses. Additionally, the time period of the observational study (October 2022–April 2023, which is the cold and flu season) increases the probability of RTIs.

### Comparison with existing literature

Several studies have demonstrated the benefits of CRP-POCTs in primary care. These benefits include reduced antibiotic prescriptions and improved treatment safety in RTIs.^1,4–7,16^ Our results align with previous studies from across Europe, which have shown positive GP perceptions towards POCTs.^10,12,13,16,29,30^ Semi-quantitative CRP-POCTs were found to be sufficient in categorising unclear symptoms and distinguishing between viral and bacterial infectious genesis. Despite the lower analytical performance of CRP-POCT,^31,32^ the interviewed GPs appeared to accept concentration ranges instead of exact numerical values. From the GPs’ perspective, semi-quantitative POCTs could be easily integrated into existing practice workflows and the concentration range gave a sufficient orientation for further procedure. CRP-POCTs were perceived as very useful owing to their simplicity, delegability, and support with communication regarding indication and performance. Rapid availability of the test result, high information gain for further diagnostics and treatment, and increased treatment safety for GPs and patients are further advantages brought by the tests. Based on the interviews with GPs, CRP-POCTs were found to be especially beneficial for older patients with multiple health conditions and for children, but current studies do not support the use of CRP-POCTs to reduce antibiotic prescriptions in children with RTIs.^33,34^ However, POCTs can alleviate stressful uncertainty among GPs and parents.

Consistent with existing literature, interviewed GPs mentioned handling and interpretation difficulties, additional time, and labour required for testing as negative aspects.^13,18,19,35^ Previous studies have confirmed that German GPs are highly willing to delegate tasks to qualified MAs.^36,37^ This also relates to the conducting of CRP-POCTs. It remains unclear whether POCTs cumulatively save or extend time. Another obstacle specific to primary care in Germany is the lack of cost-covering reimbursement. While there are positive aspects, such as subjective time and resource savings, and perceived benefits from the user’s perspective, it is important to note that cost-effectiveness has already been proven for RTIs,^38^ supporting the demand for cost-covering reimbursement.

Interestingly, interviewed GPs also used CRP-POCTs in suspected diagnoses other than RTIs. Available evidence in this regard is limited. Conducted studies lack information whether GPs diagnostic accuracy is affected and whether treatment decisions supported by CRP-POCTs improve patient safety in cases other than RTIs. Accordingly, the potential risk of the technology needs to be addressed in future implementation efforts. However, our findings are of importance for the establishment of semi-quantitative CRP-POCTs in the German primary care setting.

### Implications for research and practice

Overall, it is evident that the benefits of CRP-POCTs outweigh potential drawbacks. This aligns with the findings of the observational part of our study.^20^ In both our observational study and qualitative interviews, we demonstrated that the use of CRP-POCT enhances clinical decision making and increases the clinical confidence of GPs. Furthermore, the reasons for testing and differential diagnoses were comparable across the majority of cases, which involved not only RTIs, but also GI complaints and UTIs. Further studies are necessary to establish appropriate scenarios for the use of CRP-POCTs in general practice. Our interviews indicate that GPs do not perform shared decision making regarding the use of CRP-POCTs. Therefore, additional research is needed to incorporate the patient’s perspective.

German GPs consider semi-quantitative CRP-POCTs to be valuable diagnostic tools owing to their ability to facilitate immediate clinical decisions when compared with conventional laboratory testing. Utilisation can increase diagnostic and therapeutic certainty in decision making. The integration of these tests into practice is considered simple, with tolerable barriers to routine use. Adjusting reimbursement to cover the costs of tests could increase their utilisation among GPs in Germany. All responders expressed their willingness to continue using the tests in their practice, some of them only if provided with cost-covering reimbursement. Further studies are needed to determine the validity and objective benefits of semi-quantitative POCTs in primary care.

## Trial registration number

German Clinical Trials Register: DRKS00030333.

## Data

The dataset relied on in this article is available from the corresponding author on reasonable request.
